# Accuracy and Safety of Dexcom G7 Continuous Glucose Monitoring in Adults with Diabetes

**DOI:** 10.1089/dia.2022.0011

**Published:** 2022-05-31

**Authors:** Satish K. Garg, Mark Kipnes, Kristin Castorino, Timothy S. Bailey, Halis Kaan Akturk, John B. Welsh, Mark P. Christiansen, Andrew K. Balo, Sue A. Brown, Jennifer L. Reid, Stayce E. Beck

**Affiliations:** ^1^ Department of Medicine and Pediatrics, Barbara Davis Center for Diabetes, University of Colorado, Aurora, Colorado, USA.; ^2^ DGD Clinic, San Antonio, Texas, USA.; ^3^ Sansum Diabetes Research Institute, Santa Barbara, California, USA.; ^4^ AMCR Institute, Escondido, California, USA.; ^5^ Dexcom, Inc., San Diego, California, USA.; ^6^ Diablo Clinical Research, Walnut Creek, California, USA.; ^7^ University of Virginia, Charlottesville, Virginia, USA.

**Keywords:** Continuous glucose monitoring, Dexcom, Accuracy, G7, MARD

## Abstract

**Background:**

We evaluated the accuracy and safety of a seventh generation (G7) Dexcom continuous glucose monitor (CGM) during 10.5 days of use in adults with diabetes.

**Methods:**

Adults with either type 1 or type 2 diabetes (on intensive insulin therapy or not) participated at 12 investigational sites in the United States. In-clinic visits were conducted on days 1 or 2, 4 or 7, and on the second half of day 10 or the first half of day 11 for frequent comparisons with comparator blood glucose measurements obtained with the YSI 2300 Stat Plus glucose analyzer. Participants wore sensors concurrently on the upper arm and abdomen. Accuracy evaluation included the proportion of CGM values within 15% of comparator glucose levels >100 mg/dL or within 15 mg/dL of comparator levels ≤100 mg/dL (%15/15), along with the %20/20 and %30/30 agreement rates. The mean absolute relative difference (MARD) between temporally matched CGM and comparator values was also calculated.

**Results:**

Data from 316 participants (619 sensors, 77,774 matched pairs) were analyzed. For arm- and abdomen-placed sensors, overall MARDs were 8.2% and 9.1%, respectively. Overall %15/15, %20/20, and %30/30 agreement rates were 89.6%, 95.3%, and 98.8% for arm-placed sensors and were 85.5%, 93.2%, and 98.1% for abdomen-placed sensors. Across days of wear, glucose concentration ranges, and rates of change, %20/20 agreement rates varied by no more than 9% from the overall %20/20. No serious adverse events were reported.

**Conclusions:**

The G7 CGM provides accurate glucose readings with single-digit MARD with arm or abdomen placement in adults with diabetes. Clinicaltrials.gov: NCT04794478

## Introduction

Over the past decade, there have been several advances in diabetes technology such as continuous glucose monitoring (CGM) systems, insulin delivery devices, and hybrid closed-loop systems. CGMs are increasingly used by people with type 1 diabetes (T1D) and type 2 diabetes (T2D), especially those requiring insulin, to improve and maintain good glycemic control.^1–4^ The currently available G6 CGM (Dexcom, Inc., San Diego, CA, USA) provides accurate readings, as demonstrated in earlier studies in adult^5,6^ and pediatric^7^ populations.

The glucose values are provided at 5-min intervals and can be used for diabetes treatment decisions or can be acted upon by devices from other manufacturers, such as automated insulin delivery systems or multidose memory insulin pens. G6 features such as its optional calibrations, predictive alerts, and automated sensor insertions facilitated diabetes management when G6 was introduced >3 years ago.

A seventh generation (G7) CGM (Dexcom) was designed to improve upon the performance and usability aspects of the G6 CGM. Similar to G6, the G7 relies on a subcutaneous glucose oxidase-based sensor that is factory calibrated and allows for optional user-initiated calibrations. Data from either system can be displayed on a dedicated receiver or on a variety of iOS and Android smart devices; both the G6 and the G7 use Bluetooth low energy to transmit data with an obstacle-free range of 20 feet. However, the on-body portion of G7 is 60% smaller than that of G6, and each sensor includes its own single-use transmitter. G7 features several other differences including automatic session initializations, a 27-min warm-up period (vs. 2 h for G6), and a 12-h (half a day) grace period at the end of the sensor's 10-day working life during which glucose data are still displayed.

If the receiver detects a gap in the data, up to 24 h of missing data can be requested from the wearable portion of the system to display glucose values that were not successfully transmitted at the time of collection. G7, like G6, is not susceptible to interference by acetaminophen or ascorbic acid. Users have the option to customize settings for rate-of-change (RoC) alerts and can disable audible glucose alerts for up to 6 h. G7 also offers real-time remote monitoring (“Sharing”) functionality, which caregivers and providers may find useful.

This multicenter study evaluated the accuracy and safety attributes of G7 in adults with diabetes. The performance of sensors worn on the arm and abdomen was assessed across the 10.5-day wear period, different glucose concentration ranges, and various rates of glucose changes. Adverse events related to sensor insertion, wear, and removal were also reported.

## Methods

### Study design and participants

This prospective multicenter single-arm study was conducted at 12 sites in the United States. The protocol and consent forms were approved by local and/or centralized institutional review boards. Written informed consent was obtained from each study participant before study initiation.

**FIG. 1.**
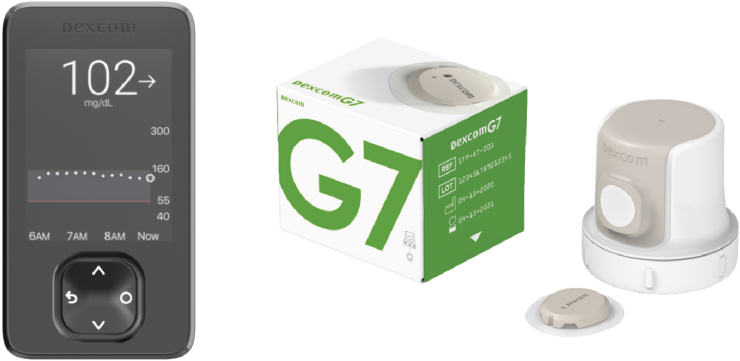
G7 components (left to right) include an optional receiver and display unit, packaging, the wearable sensor/transmitter, and the applicator. The wearable measures 24 × 27.3 × 4.6 mm, roughly the size of three stacked quarters. G7, seventh generation.

Between February and June 2021, the study enrolled 318 adults of ages 18–78 years. Participants were eligible if they had T1D, intensive insulin therapy T2D (T2D-IIT), or nonintensive insulin therapy T2D (T2D-NIIT). Participants were recruited without regard to race, gender, or body mass index (BMI). Inclusion criteria included willingness to wear up to three G7 devices for the duration of the study, and willingness to avoid injecting insulin and wearing an insulin pump infusion set within 3 inches of the insertion site. G7 components are shown in Figure 1.

The wearable transmitter measures 24 × 27.3 × 4.6 mm, or roughly the size of three stacked quarters. All subjects participated in clinic sessions involving venous blood draws. Exclusion criteria included the presence of skin conditions at sensor wear sites, known allergy to medical-grade adhesives, pregnancy, abnormal hematocrit levels, and medical conditions that could compromise patient safety or staff safety (at the investigator's discretion). Detailed inclusion and exclusion criteria are provided in Supplementary Table S1. The study protocol was reviewed by the Food and Drug Administration through the investigational device exemption process and registered at (clinicaltrials.gov).

### Study procedures

Each participant wore G7 sensors on the back of the upper arm and on the abdomen. Neither device provided real-time glucose concentrations or alerts to the patients or the research staff during the study. Participants and research teams were not provided with the optional receiver/display component shown in Figure 1. After participants received training on G7, sensor insertions were performed in the clinic by the participants. G7 CGMs that failed within the first 12 h were replaced in the same wear location. Participants completed surveys about the ease of G7 insertion and preferred device wear location.

During home use, participants were advised to continue their usual glucose monitoring practices (blood testing or use of a personal CGM), diabetes management regimens, and activities. Participants were also scheduled for three 12-h clinic sessions on days 1 or 2, days 4 or 7, and the second half of day 10 (hours 228 to 240) or first half of day 11 (hours 240 to 252) of the wear period. During clinic sessions, arterialized venous blood glucose concentrations were determined with the YSI 2300 Stat Plus glucose analyzer (YSI, Inc., Yellow Springs, OH, USA).

Hyperglycemia and hypoglycemia were induced by adjusting the timing or amount of rapid-acting insulin and carbohydrates; the amount of insulin or carbohydrates was based on participants' usual insulin dosing and meal timing and authorized by the clinical research staff. These per-protocol manipulations were done under close observation by the clinical investigational staff. Blood glucose determinations were made at 15 ± 5-min intervals for YSI values in the 80–300 mg/dL range and at 10 ± 5-min intervals for YSI values that were <80 or >300 mg/dL.

All CGM removals were performed by participants. For participants assigned to a clinic session on the second half of day 10 or first half of day 11, G7 removals were performed after completion of the clinic session. During the removal visit, study staff evaluated insertion sites and adhesive areas, and documented any participant-reported adverse events.

### Data analysis

To obtain CGM-measured glucose values for analysis, raw sensor signals collected by the transmitter were postprocessed using a proprietary algorithm. YSI measurements taken during the clinic sessions were matched with the first available CGM value obtained within the subsequent 5 min, including only CGM values within the 40–400 mg/dL reportable range. Sensors that failed before a subject's first clinic session were excluded from the analysis.

Accuracy metrics included %15/15 (the proportion of CGM values that were within 15% of paired YSI values >100 or 15 mg/dL of YSI values ≤100 mg/dL), as well as %20/20 and %30/30 agreement rates, the mean absolute difference (MAD), and the mean absolute relative difference (MARD) of each CGM-YSI pair. MAD is given in units of glucose concentration and is appropriate for YSI values in the low range, where relative differences may be large. For higher YSI values where absolute differences may be large, MARD is given as a percent and is calculated as the mean ratio of the absolute difference between each CGM-YSI pair and the YSI value.

For analysis of accuracy across CGM glucose ranges, MAD is presented for CGM values ≤80 mg/dL and MARD is presented for CGM values >80 mg/dL. Sensor heterogeneity was assessed by analyzing each sensor's MARD and by determining the proportion of sensors having >80% of their values meet the %20/20 accuracy criterion. Performance was assessed across glucose concentration ranges and days of sensor wear. Heterogeneity of accuracy within patient subgroups was analyzed using a generalized estimating equations model to adjust for intrasubject correlation.

Accuracy was also analyzed at various CGM rates of glucose concentration change. RoC was calculated as the difference between consecutive glucose values per unit of time, in units of mg/dL per minute. MARD and %20/20 were evaluated for the following RoC categories: <−2, −2 to <−1, −1 to <0, 0 to 1, >1 to 2, and >2.

G7 detection of hypoglycemia and hyperglycemia was evaluated by identifying CGM values surrounding YSI values in <70 and >250 mg/dL ranges. The analyses were conducted assuming that the G7 low and high alert thresholds were set to 55 and 300 mg/dL, respectively. True hypoglycemia alerts were those accompanied within 15 min by YSI value(s) <70 mg/dL; similarly, true hyperglycemia alerts were those accompanied by YSI value(s) >250 mg/dL.

Separately, more stringent criteria for hypoglycemic and hyperglycemic alerts and detections were applied. For alerts, YSI values within 15 min of an out-of-range CGM value were considered, and for detections, CGM values within 15 min of an out-of-range YSI value were considered. For the hypoglycemic alert rate, a CGM value below a specific threshold was considered a hypoglycemic alert; it was considered a true alert if at least one YSI value within the 15-min window was also below the specified threshold. For the hyperglycemic alert rate, a CGM value above a specific threshold was considered a hyperglycemic alert; it was considered a true alert if at least one YSI value within the 15-min window was also above the specified threshold.

For the hypoglycemic detection rate, a YSI value below a specific threshold was considered a hypoglycemic event; it was considered a true detection if at least one CGM value within the 15-min window was also below the specified threshold. For the hyperglycemic detection rate, a YSI value above a specific threshold was considered a hyperglycemic event; it was considered a true detection if at least one CGM value within the 15-min window was also above the specified threshold. G7 Urgent Low Soon alerts, identical to those of G6, were triggered by downward trends in CGM values predicted to progress to CGM value(s) ≤55 mg/dL within the next 20 min.

To estimate lag time for each sensor, CGM measurements were linearly interpolated to estimate glucose values at 1-min intervals. The MARD was calculated for every minute of lag time for each sensor, and the lag time associated with the smallest MARD was assigned as the lag time for that sensor. Mean lag time was calculated for all sensors, and for sensors placed at each wear location.

Device safety was assessed by evaluation of all device-related adverse events. All analyses were performed using SAS software, version 9.4 (SAS Institute, Inc., Cary, NC, USA).

## Results

### Study population

Baseline demographics are summarized in Table 1. The study enrolled 318 adult participants. More than two-thirds of the participants were overweight or obese, consistent with the prevalence of these conditions among people with diabetes in the United States. The participants attempted to initialize 670 sensors, and of these, 40 failed to return any glucose values. One participant withdrew from the study before YSI measurements, and one did not qualify for clinic sessions due to an associated comorbidity of uncontrolled hypertension. The remaining 316 participants provided data from 308 arm and 311 abdomen devices (79,003 CGM-YSI matched pairs). Of these matched pairs, there were 77,774 in the reportable range of 40–400 mg/dL (39,193 from arm devices and 38,581 from abdomen devices) that were included in the calculation of accuracy metrics.

**Table 1.** Baseline Participant Characteristics (*N* = 318)

Demographic	Value
Age, years	
Mean (SD)	44.3 (15.7)
Median	44.3
Min, max	18.1, 78.3
Diagnosis, *n* (%)	
T1D	257 (80.8)
T2D-IIT	51 (16.0)
T2D-NIIT	10 (3.1)
Gender, *n* (%)	
Female	170 (53.5)
Male	148 (46.5)
Ethnicity, *n* (%)	
Hispanic or Latino	78 (24.5)
Not Hispanic or Latino	240 (75.5)
Race, *n* (%)	
American Indian or Alaska Native	1 (0.3)
Asian	4 (1.3)
Black; African American; or of African heritage	16 (5.0)
Native Hawaiian or Pacific-Islander	2 (0.6)
White	286 (89.9)
Other	9 (2.8)
BMI category, *n* (%)	
Underweight (<18.5 kg/m^2^)	1 (0.3)
Normal (18.5–<25 kg/m^2^)	105 (33.0)
Overweight (25–<30 kg/m^2^)	92 (28.9)
Obese Class I (30–<35 kg/m^2^)	70 (22.0)
Obese Class II (35–<40 kg/m^2^)	33 (10.4)
Obese Class III (≥40 kg/m^2^)	17 (5.3)
BMI, kg/m^2^	
Mean (SD)	28.9 (6.3)
Median	28.1
Min, max	17.4, 54.0

BMI, body mass index; SD, standard deviation; T1D, type 1 diabetes; T2D, type 2 diabetes; T2D-NIIT, nonintensive insulin therapy T2D; T2D-IIT, intensive insulin therapy T2D.

### Overall MARD and agreement rates

The overall MARD for arm-placed sensors was 8.2%, and the overall %15/15, %20/20, and %30/30 agreement rates were 89.6%, 95.3%, and 98.8%, respectively. Similarly, the overall MARD for abdomen-placed sensors was 9.1%, and the overall %15/15, %20/20, and %30/30 were 85.5%, 93.2%, and 98.1%, respectively.

### Individual sensor accuracy

**FIG. 2.**
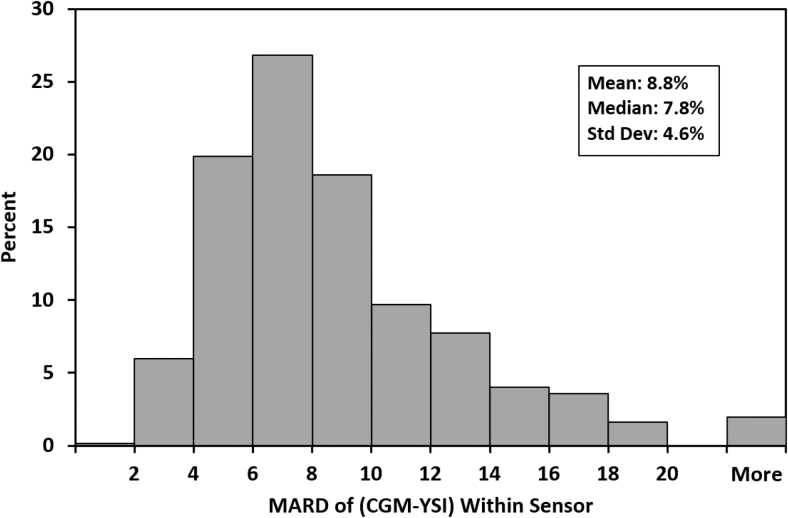
Aggregated sensor MARD (%) of CGM-YSI histogram plot for 619 sensors placed on the arm or abdomen. CGM-YSI; MARD, mean absolute relative difference.

Among the 308 sensors worn on the arm, 291 (94.5%) had >80% of CGM-YSI matched pairs that met the %20/20 accuracy criterion. Similarly, among the 311 sensors worn on the abdomen, 272 (87.5%) had >80% of matched pairs that met the %20/20 accuracy criterion. Figure 2 summarizes the performance of all 619 individual sensors. The mean and median per-sensor MARDs were 8.8% and 7.8%, respectively, 442 (71.4%) had MARD values <10%, and 12 (1.9%) had MARD values >20%.

### Accuracy across days of wear, glucose ranges, and patient characteristics

Accuracy across clinic days is summarized in Table 2, and accuracy across glucose ranges is summarized in Table 3. In Table 3, MAD is presented in the 40–60 and 61–80 mg/dL ranges; MARD is presented for ranges >80 mg/dL. Accuracy by diabetes type and insulin regimen is summarized in Table 4. Accuracy remained high in both arm- and abdomen-placed sensors across the 10-day wear period through the 12-h grace period, and across glucose ranges. There were no statistically significant differences between G7 accuracy when participants were analyzed by diabetes type and insulin regimen.

**Table 2.** Sensor Accuracy Across Days of Sensor Wear

Placement	Clinic session day	Matched pairs (*n*)	%15/15 (%)	%20/20 (%)	%30/30 (%)	MARD (%)
Arm (*N* = 308)	Day 1	6001	76.9	87.4	95.9	11.9
	Day 2	8279	89.4	95.5	98.8	8.4
	Day 4	7312	91.8	97.2	99.6	7.2
	Day 7	5898	93.7	97.5	99.5	7.2
	Day 10	5787	92.4	96.4	99.4	7.6
	Day 10.5	5916	93.2	97.4	99.4	6.9
Abdomen (*N* = 311)	Day 1	5864	73.0	84.5	94.7	12.9
	Day 2	8438	86.5	94.9	99.3	8.6
	Day 4	7624	90.9	96.6	99.3	7.7
	Day 7	6088	89.5	95.3	98.5	8.1
	Day 10	5828	84.5	92.9	98.2	9.3
	Day 10.5	4739	86.4	93.1	97.6	8.8

MARD, mean absolute relative difference.

**Table 3.** Sensor Accuracy Across Continuous Glucose Monitoring Glucose Ranges

Placement	Glucose range (mg/dL)	Matched pairs (*n*)	%15/15 (%)	%20/20 (%)	%30/30 (%)	MAD (mg/dL)	MARD (%)
Arm (*N* = 308)	40–60	2444	85.1	91.9	97.0	8.5	NA
	61–80	5485	92.6	96.5	98.9	6.3	NA
	81–180	15,319	86.2	93.6	98.3	NA	8.9
	181–300	10,465	90.3	96.0	99.3	NA	7.2
	301–400	5480	96.8	99.1	99.9	NA	5.4
Abdomen (*N* = 311)	40–60	2436	77.1	85.0	94.2	10.3	NA
	61–80	5309	89.4	94.1	97.8	7.3	NA
	81–180	15,074	82.7	92.0	97.7	NA	9.6
	181–300	10,108	85.1	93.4	98.8	NA	8.3
	301–400	5654	93.5	98.6	99.8	NA	6.2

MAD, mean absolute difference; NA, not applicable.

**Table 4.** Sensor Accuracy by Diabetes Type and Insulin Regimen

Placement	Diabetes type and insulin regimen	No. of subjects	No. of matched pairs	%15/15 (%)	%20/20 (%)	%30/30 (%)	MARD (%)
Arm (*N* = 308)	Type 1	252	31,948	89.8	95.4	98.8	8.2
	Type 2—IIT	46	6005	88.8	94.7	98.7	8.0
	Type 2—NIIT	10	1240	87.7	96.0	99.0	9.2
Abdomen (*N* = 311)	Type 1	253	31,109	85.0	92.5	97.9	9.3
	Type 2—IIT	48	6260	87.6	95.8	99.1	8.6
	Type 2—NIIT	10	1212	87.8	97.2	99.6	7.6

IIT, intensive insulin therapy; NIIT, nonintensive insulin therapy.

Heterogeneity of accuracy was also assessed across the subgroups of gender (male/female) and BMI category (underweight/normal, overweight, obese Class I, obese Class II/III) as defined in Table 1. There were no statistically significant differences in accuracy (results not shown) across these subgroups. Participants in the lowest BMI decile (<22 kg/m^2^) had a %20/20 agreement rate and MARD of 95.3% and 7.8%, respectively; those in the nine higher BMI deciles (≥22 kg/m^2^) had a %20/20 agreement rate and MARD of 94.1% and 8.8%, respectively.

### Accuracy at various RoCs

G7 accuracy at different glucose concentration RoCs is summarized in Table 5. The highest %20/20 agreement rates and lowest MARDs occurred when CGM readings were increasing or decreasing by no more than 1 mg/dL per minute. For arm-placed sensors, the MARDs at rapidly decreasing (RoC <−2 mg/dL per minute) or rapidly increasing (RoC >2 mg/dL per minute) were 9.3% and 9.7%, respectively, and the %20/20 agreement rates were 90.1% and 90.3%, respectively. Similarly, for abdomen-placed sensors, MARDs at rapidly decreasing or increasing ROCs were 10.4% and 10.3%, respectively, and %20/20 agreement rates were 87.9% and 89.4%, respectively.

**Table 5.** Sensor Accuracy at Various Rates of Glucose Concentration Change

Placement	CGM rate range (mg/dL per minute)	Matched pairs (*n*)	%20/20 (%)	MARD (%)
Arm (*N* = 308)	<−2	1006	90.1	9.3
	−2 to <−1	3266	94.0	8.4
	−1 to <0	15,220	96.2	8.1
	0 to 1	12,657	96.5	7.8
	>1 to 2	3742	94.3	8.3
	>2	2411	90.3	9.7
Abdomen (*N* = 311)	<−2	895	87.9	10.4
	−2 to <−1	2997	91.4	9.3
	−1 to <0	15,350	94.3	9.0
	0 to 1	12,486	93.6	9.0
	>1 to 2	3599	93.5	8.7
	>2	2337	89.4	10.3

CGM, continuous glucose monitoring.

### Threshold and Urgent Low Soon alert performance

When the hypoglycemia threshold alert was set to 55 mg/dL, the true alert rates for detection of hypoglycemia <70 mg/dL by sensors worn on the arm and abdomen were 91.3% and 85.2%, respectively. When the hyperglycemia threshold alert was set to 300 mg/dL, the true alert rates for detection of hyperglycemia >250 mg/dL by sensors worn on the arm and abdomen were 99.9% and 99.8%, respectively. Table 6 summarizes G7 alert and detection rates for hypoglycemia and hyperglycemia at various thresholds using more stringent criteria.

**Table 6.** Hypoglycemic and Hyperglycemic Alerts and Detections at Various Thresholds

Condition	Placement	Alert or detection level (mg/dL)	No. of alerts	True alert rate (%)	No. of detections	True detection rate (%)
Hypoglycemia	Arm (*N* = 308)	55	2189	51.0	1037	75.8
		70	7339	86.9	5651	88.8
		90	16,749	92.2	10,674	96.1
	Abdomen (*N* = 311)	55	2498	45.9	1046	73.3
		70	7554	82.1	5648	87.6
		90	16,653	90.4	10,637	95.0
Hyperglycemia	Arm (*N* = 308)	120	56,899	97.0	24,147	98.5
		180	35,465	96.4	16,454	97.8
		200	29,941	96.3	14,521	97.1
		240	20,970	95.6	11,244	95.9
		300	8884	90.1	6630	88.7
	Abdomen (*N* = 311)	120	56,013	97.0	23,854	98.0
		180	35,401	95.9	16,295	97.2
		200	30,040	95.5	14,380	96.8
		240	21,041	94.0	11,139	94.4
		300	9636	84.2	6538	86.8

All participants were considered in the analysis; however, not all participants experienced a hypoglycemic or hyperglycemic event.

Of the 17 Urgent Low Soon alerts arising from arm-placed sensors, 8 (47.1%) were followed within 30 min by YSI value(s) ≤55 mg/dL and 16 (94.1%) were followed within 30 min by YSI value(s) ≤70 mg/dL. Of the 27 Urgent Low Soon alerts arising from abdomen-placed sensors, 11 (40.7%) were followed within 30 min by YSI value(s) ≤55 mg/dL and 18 (66.7%) were followed within 30 min by YSI value(s) ≤70 mg/dL.

### Time lag

The overall mean (standard error [SE]) time lag for the 619 sensors worn in the study was 3.5 (0.13) min. The mean (SE) time lags for sensors worn on the arm and abdomen were 3.6 (0.20) min and 3.4 (0.19) min, respectively.

### Adverse events and user experience

There were no serious adverse events in the study population. Four participants reported mild to moderate device-related adverse events. One participant experienced moderate erythema at the insertion site (arm) and adhesive areas, one experienced mild skin tearing at the adhesive area (abdomen), one experienced moderate erythema at the adhesive area (abdomen) as well as mild discomfort during sensor removal, and one experienced moderate discomfort during sensor removal (arm). A total of 241 participants (75.8%) had experience with a personal CGM, and of these, 164 (68.1%) found G7 easier to insert than their current personal device. Of the participants who expressed a preference for wear location, most (51.7%) indicated a preference for arm wear.

## Discussion

The G7 CGM described in this pivotal study was accurate and safe when inserted on either the upper arm or abdomen in adults with diabetes. In-clinic glucose manipulations and frequent blood glucose sampling confirmed accurate readings during euglycemia, hypoglycemia, and hyperglycemia (reflected as time in range [TIR], time below range [TBR], and time above range [TAR]), as well as during rapid glucose concentration change. Even at the highest rates of glucose concentration change, MARD values <10% were observed for arm-placed sensors and were <10.5% for abdomen-placed sensors. There were no safety concerns during the study.

The MARD values of 8.2% (arm) and 9.1% (abdomen) were similar to or better than accuracy measurements of other commercially available CGM systems.^8–10^ Few studies have directly compared CGM accuracy at different anatomical locations. For example, Steineck et al.,^11^ in a study of the G4 Platinum system (Dexcom), reported MARD estimates of 12.0% for arm-placed and 12.3% for abdomen-placed sensors, suggesting slightly better accuracy for the arm. In a study of pregnant women with diabetes using the G6 system,^12^ the MARD values for sensors worn on the arm, abdomen, and buttock were 8.7%, 11.5%, and 11.2%, respectively.

An earlier in silico study suggested that MARD values <10% allow for insulin dosing decisions without reliance on blood glucose test results.^13^ In this study, single-digit MARD values were observed for >70% of the individual sensors. The prevalence of sensors with MARD values ≥20% was lower than was observed in an earlier accuracy study of G6 sensors^6^; the basis of anomalously poor accuracy in a small proportion of sensors is unknown. Consistent with earlier studies of systems from Dexcom and other manufacturers, slightly lower accuracy was observed immediately after sensor insertion,^14^ during hypoglycemia,^15^ and at rapid rates of glucose concentration change.^16^

Time delays between perturbations in YSI and G7 readings were also similar to or better than time delays in prior generation Dexcom CGMs. In 2009, Bailey et al.^17^ estimated the lag time of Dexcom's SEVEN Plus system as 11 min, and in 2020, Guillot et al.^18^ reported a lag time of 13 min for G6 during exercise. Using data from the pivotal accuracy study, Calhoun et al.^19^ estimated the G6 lag time as 3.7 min, and noted that 23% of the G6 sensors had lag times of <1 min. The mean lag time of 3.5 min reported here for G7 is likely attributable to improvements in both the hardware and signal processing algorithm and may have implications for automated insulin delivery systems.

Several aspects of G7 hardware and software are different from G6. The G7 sensor wire is shorter than that of G6 and is inserted at a steeper (90°) angle. The redesigned applicator allows for sensor deployment with one hand, and most participants found G7 easier to insert than their prior CGM system. Integration of the sensor and transmitter eliminates the need for using a transmitter across multiple sensor sessions (as is the case for G6).

The G7 adhesive patch is smaller, about half the size of the G6 adhesive patch. In addition, data are available from G7 within 27 min of insertion versus a 2-h startup time for G6, and G7 data continue to be available for a 12-h “grace period” at the end of a sensor session. As is the case with G6, calibration of G7 sensors remains optional. Unlike G6, G7 allows for temporary silencing of all audible alerts, including Urgent Low. Taken together, these attributes are anticipated to provide for a better end-user experience with G7 and help reduce diabetes burden.

The main strength of the study was the large number of devices, participants, and investigational sites, which provided many matched pairs. An additional strength was the in-clinic glucose manipulations that allowed for accuracy determinations in different glycemic ranges and at various rates of glucose concentration change. The study also allowed device placement on the abdomen or arm, which reflects real-world placement practices. The preference for arm over abdomen wear observed here and in an earlier report of Instagram photographs^20^ may reflect disadvantages of abdomen wear such as jostling or compression by clothing or seat belts.

The study excluded children and adolescents; data from this population will be reported separately. Participants were predominantly White, and two-thirds were overweight or obese, which is consistent with attributes reported for adult T1D Exchange registry participants^21^ and with the high prevalence of overweight and obesity in adults with T2D. The study was not designed to evaluate clinical outcomes such as HbA1c reduction, or changes to metrics such as TIR, TBR, and TAR. Because the alert functions were disabled during the study, real-world alert performance attributes (especially for the Urgent Low Soon alert) may differ from the rates reported here. Performance and outcomes will be evaluated in subsequent trials and retrospective analyses of real-world use.

## Conclusion

This study demonstrates that the G7 CGM is accurate and safe to use for up to 10.5 days in adults with diabetes when worn on either the arm or abdomen. G7 is significantly smaller than G6 (by 60%) and introduces new features such as sensor/transmitter integration with a simplified insertion process. The enhanced features of G7 may increase clinical adoption, encourage sustained use, and reduce the burden of diabetes management.

## Supplementary Material

Supplemental data
