# Supplementary material for: Accuracy and Safety of Dexcom G7 Continuous Glucose Monitoring in Adults with Diabetes
Source: Diabetes Technol Ther. 2022 May 31;24(6):373–80. doi: 10.1089/dia.2022.0011 (PMC9208857; doi:10.1089/dia.2022.0011)
Supplement: Supplemental data [file Supp_TableS1.docx]

Supplemental Material for Satish Garg et al., “**Accuracy and Safety of Dexcom G7 Continuous Glucose Monitoring in Adults with Diabetes**”

Table S1: Study Eligibility Criteria

| **Inclusion Criteria** | **Exclusion Criteria** |
| --- | --- |
| Subjects may be included if they meet all of the following criteria:  1. Ages ≥ 18 years (“age” determined at the time of the Insertion Visit) with either of the following:   1. Diagnosis of type 1 diabetes (T1D) or type 2 diabetes (T2D) and on intensive insulin therapy (IIT) with known dosing parameters for at least three (3) months prior to the Screening Visit. 2. Diagnosis of type 2 diabetes (T2D) and on non-intensive insulin therapy (NIIT). **Note:** No more than 10 subjects of ages ≥ 18 years will be on non-intensive insulin therapy (NIIT).    1. 2. Weigh at least 110 lbs. (50 kilograms).    2. 3. Willing to wear up to three (3) Systems for the total duration of study wear [up to 10.5 days of total wear (252 hours) + 48 hours for scheduling purposes].    3. 4. Willing to avoid injecting insulin and wearing an insulin pump infusion set within three (3) inches of the System insertion site(s).    4. 5. Is able to speak, read, and write in English.    5. 6. Willing to participate in up to three (3) Clinic Sessions: Each Clinic Session will be approximately twelve (12) hours (maximum of 13 hours) in duration, during which frequent venous sampling (not to exceed 3 mL/kg) and SMBG testing will be performed. Subjects on intensive insulin therapy (IIT) will participate in interventional clinic sessions with glucose excursions, during which deliberate insulin and glucose challenges will be performed to induce hyperglycemia and hypoglycemia. There will be no explicit attempt to manipulate glucose levels for subjects on non-intensive insulin therapy (NIIT). **Note:** The amount of blood samples taken per Clinic Session will vary according to the subject’s weight and age at the Insertion Visit and the maximum Estimated Blood Loss (EBL). | Subjects will be excluded if they meet any of the following criteria:  1. Presence of extensive skin changes/diseases at sensor wear site(s) that preclude wearing the sensor(s) on skin (e.g. extensive psoriasis, recent burns or severe sunburn, extensive eczema, extensive scarring, extensive tattoos, or dermatitis herpetiformis).  2. Known allergy to medical-grade adhesives.  3. For female subjects of child-bearing potential: Pregnancy, demonstrated by a positive test no more than 72 hours prior to the Insertion Visit, and not willing to use an acceptable form of contraception during study wear.  4. Have donated blood, had significant blood loss, or participated in a study with significant blood sampling within 56 days prior to study enrollment or plan to partake in such activities during study wear.  5. Hematocrit measurement via point-of-care (POC) or laboratory testing that is less than the applicable below-mentioned value:  • Male: 36.0%  • Female: 33.0%  6. For those on intensive insulin therapy (IIT), use of bolus U-500 insulin in routine diabetes care.  7. For those on intensive insulin therapy (IIT), any of the following medical history:  • Cardiovascular disease including, but not limited to the following conditions: ischemic heart disease, peripheral vascular disease, cardiomyopathy, cerebrovascular disease, congenital heart disease, significant arrhythmias, or use of a pacemaker  • Epilepsy  • Syncope within the past 12 months  • Severe migraines within the past 6 months  • Adrenal insufficiency  • Significant hypoglycemia unawareness (by history)  • Diabetic ketoacidosis (DKA) event [defined as the development of ketones with diagnosis or intervention from a health care professional (i.e. EMT assistance, emergency room visit, or hospital admission)] within the last 6 month  • History of severe hypoglycemia [complicated by seizure(s) or loss of consciousness] within the last 6 months  8. End stage renal disease and currently managed by dialysis or anticipating initiating dialysis during the study wear period  9. Required or scheduled to have a Magnetic Resonance Imaging (MRI) scan, Computed Tomography (CT) scan, or diathermy during the study wear period  10. Current participation in another investigational study protocol (If a subject has recently completed participation in another drug study, the subject must have completed that study at least 30 days prior to being enrolled in this study). Note: Subjects will not be excluded if enrolled in another observational trial, wherein the subject is in the follow-up phase and no tests/procedures impacting the subject’s health are required. Subjects will be excluded if they have been previously enrolled in this study  11. The subject and/or immediate family member(s), and/or person(s) living within the household work for Dexcom, Medtronic, GlySens Inc., Abbott Laboratories, Roche, Senseonics, Waveform, Ascensia Diabetes Care, or POCTech;  12. Any condition that, in the opinion of the Investigator, would interfere with their participation in the trial or pose excessive risk to study staff. Some examples of conditions that Investigators may consider exclusionary are HIV, Hepatitis B, or Hepatitis C infections; active illegal substance use; or active psychiatric disorder. |
